# Sinogenic Subdural Empyema in an Adolescent: Multifocal Disease, Decompressive Craniotomy, and Complete Recovery

**DOI:** 10.7759/cureus.115856

**Published:** 2026-09-06

**Authors:** Ricardo G Blanco-Ortiz, Alejandro Arbona-Lampaya, Edwin J Medina González, Shermain Aponte Rosario, Alejandra Diaz-Rohena, Mayra Vizcarrondo

**Affiliations:** 1 School of Medicine, University of Puerto Rico, Medical Sciences Campus, San Juan, PRI; 2 Pediatrics, University of Puerto Rico, Medical Sciences Campus, San Juan, PRI

**Keywords:** decompressive craniotomy, endoscopic sinus surgery, intracranial complications of sinusitis, pediatric neurosurgery, subdural empyema

## Abstract

Sinogenic subdural empyema is a rare and life-threatening complication of bacterial sinusitis. Non-specific symptoms tend to make early diagnosis and management challenging. We report the case of a previously healthy 16-year-old male who presented with a one-week history of upper respiratory symptoms and headache followed by a rapidly progressive neurologic deterioration. Multifocal subdural empyema was confirmed via multiparametric MRI. Cultures taken during neurosurgical intervention identified a polymicrobial etiology, including *Staphylococcus aureus*,* Staphylococcus epidermidis*,* Streptococcus constellatus*,and *Streptococcus intermedius*, with the *Streptococcus *speciesbeing resistant to ceftriaxone and azithromycin. He underwent bifrontal craniotomies and endoscopic sinusotomies, followed by decompressive craniotomies for postoperative edema. In addition, he completed 59 days of culture-directed intravenous antimicrobial therapy. The patient recovered to his neurologic baseline. This case demonstrates the diagnostic pitfalls of early sinogenic subdural empyema in an adolescent, the role of multiparametric MRI over CT when there is suspected involvement of the intracranial space, and the potential for complete neurologic recovery despite a fulminant presentation requiring staged operative management.

## Introduction

Acute bacterial sinusitis is among the most common pediatric infections, yet in fewer than 5% of cases it escapes the paranasal compartment to produce orbital, intracranial, or osseous complications [1,2]. When intracranial extension occurs, it may manifest as meningitis, cerebritis, parenchymal abscess, epidural abscess, or subdural empyema; of these, subdural empyema is the most rapidly evolving and historically the most lethal [3,4]. Mortality in the modern era ranges from 5% to 15%, and neurologic sequelae are reported in up to one-third of survivors [4,5].

Pubertal expansion of the frontal sinus, the rich diploic and emissary venous network of the developing skull, and the absence of fibrous adhesions along the parafalcine subdural surface together favor anterograde and venous spread of infection from the frontal and ethmoid sinuses into the subdural space [6-8]. This could contribute to the over-representation of adolescents in contemporary cohorts [8]. Because the subdural compartment offers minimal anatomic resistance, pus can disseminate rapidly along the convexity and interhemispheric fissure, producing mass effect, cortical thrombophlebitis, and seizures within hours [3,9]. Early symptoms, such as headache, low-grade fever, and behavioral changes, overlap with uncomplicated viral illness, and non-contrast computed tomography is insensitive to thin subdural collections; magnetic resonance imaging with multiparametric sequences remains the diagnostic standard [10-12].

Once recognized, sinogenic subdural empyema is a neurosurgical emergency requiring urgent decompression, source control of the originating sinusitis, and prolonged broad-spectrum antimicrobial therapy [13,14]. Outcomes are believed to depend on the time from symptom onset to definitive intervention, the depth of preoperative encephalopathy, and the coordinated participation of neurosurgery, otolaryngology, infectious disease, and pediatric critical care services [5,15]. We report the case of a previously healthy 16-year-old who presented with rapidly progressive encephalopathy from multifocal subdural empyema secondary to pansinusitis. He required neurosurgical intervention, including a bifrontal and left temporal decompressive craniotomy for refractory cerebral swelling, combined left frontal, anterior, and posterior ethmoidal, and maxillary sinusotomies, and a long course of antibiotic therapy. The patient ultimately recovered to baseline neurologic function. The case illustrates both the diagnostic pitfalls of early disease and the discordance that may exist between a fulminant presentation, a complicated operative course, and an ultimately favorable recovery.

## Case presentation

A previously healthy 16-year-old male presented to the Puerto Rico Pediatric Hospital Emergency Department with altered mental status. The week prior, the patient developed symptoms of upper respiratory infection, which led him to be promptly evaluated by a pediatrician. At the time, both influenza and COVID-19 tests were negative; symptomatic treatment led to no improvement. Two days before presentation, the patient developed a severe headache, prompting a non-contrast CT, which was obtained at an outside hospital; he was then discharged on oral azithromycin and corticosteroids. One day before presenting at our ED, the patient developed confusion and incoherent speech. Review of the outside CT at the time revealed bilateral 4 mm extra-axial collections along the anterior frontal convexities, along with complete opacification of the left ethmoid and maxillary sinuses (Figures 1A-1D, 2), which were concerning for intracranial extension of sinusitis, prompting transfer to our institution.

**Figure 1:**
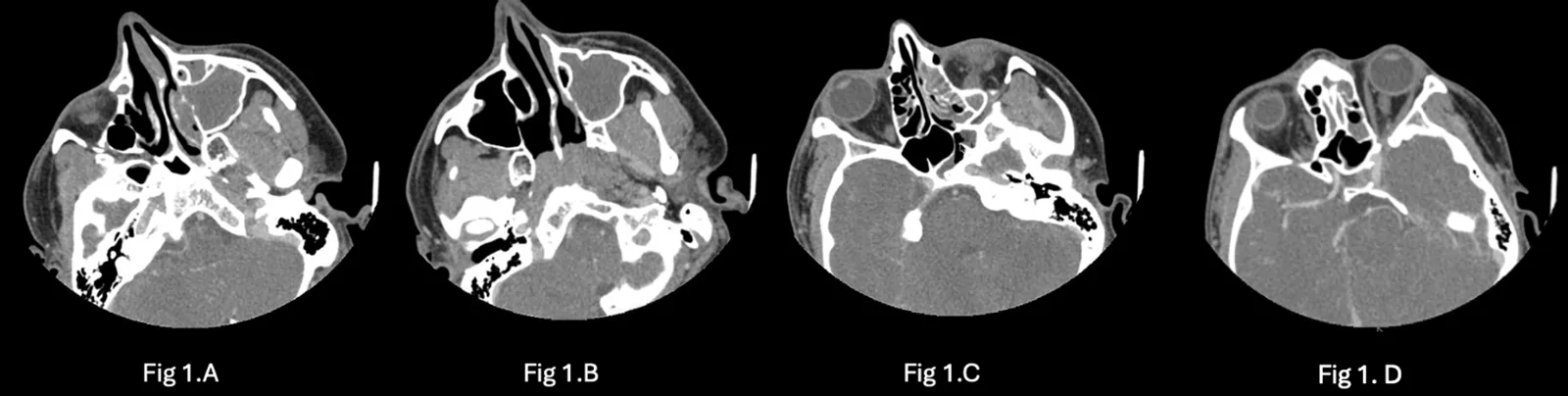
Axial CT of the paranasal sinuses demonstrating opacification of the left maxillary and ethmoidal sinuses. Non-contrast axial computed tomography of the paranasal sinuses across four sequential levels, obtained at the referring institution two days prior to transfer (A)-(D). There is complete opacification of the left maxillary sinus and left ethmoid air cells by soft-tissue density, consistent with acute bacterial sinusitis. In the context of the patient's clinical deterioration, these findings raised suspicion for intracranial extension of the sinogenic infection.

**Figure 2:**
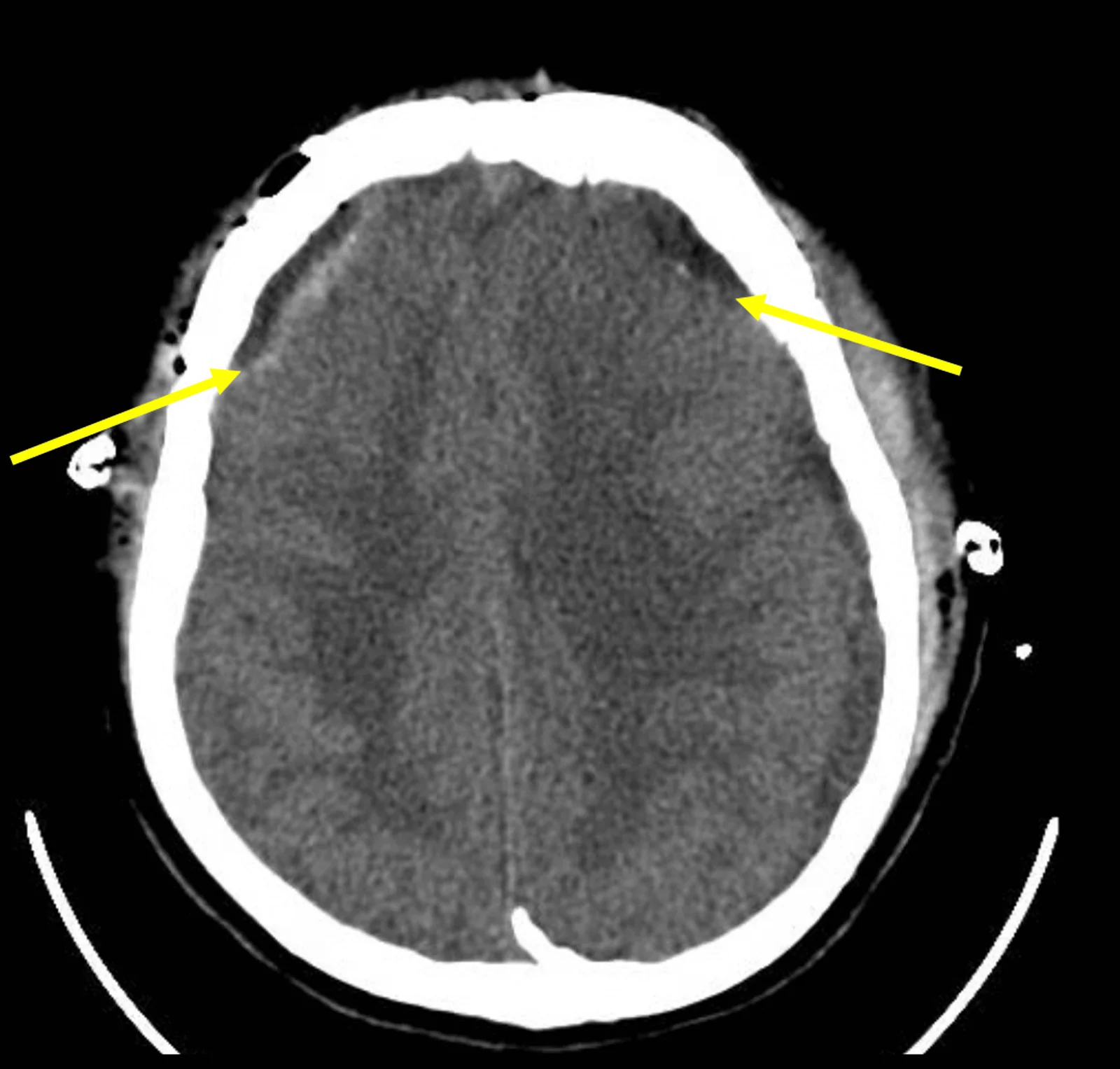
Axial CT of the brain showing bilateral frontal subdural collections. Non-contrast axial computed tomography of the head at the level of the cerebral convexity, obtained at the referring institution two days prior to transfer. Subtle bilateral, hypodense, crescentic extra-axial collections measuring 4 mm (yellow arrows) are present along the anterior frontal convexities, consistent with early subdural fluid accumulation. No midline shift is identified. The limited conspicuity of these findings on non-contrast CT underscores the modality's restricted sensitivity for thin subdural collections and the consequent risk of underestimating disease burden when magnetic resonance imaging is not obtained.

Upon arrival, the patient was incoherent and had a waxing and waning mental status, oscillating between agitation and poor arousability. He was febrile at 38.1°C and no longer following commands. At the time, laboratory results showed leukocytosis with neutrophilia and anemia (Table 1). Multiparametric MRI confirmed multifocal subdural empyema, and empiric therapy with vancomycin, ceftriaxone, and metronidazole was initiated (Figures 3, 4).

**Table 1:** Hematologic trends during the acute phase of hospitalization (hospital days 0 to 4). Hematologic laboratory trend during the acute phase of hospitalization (hospital days 0-4). Marked leukocytosis with neutrophilic predominance and a left shift on admission declined following combined neurosurgical evacuation and endoscopic sinus source control. The erythrocyte sedimentation rate, measured on hospital day 2, was markedly elevated at 106 mm/h. Concurrent normocytic anemia was present throughout, consistent with anemia of acute inflammation. H, above reference range; L, below reference range; -, not measured; ANC, absolute neutrophil count; ESR, erythrocyte sedimentation rate; WBC, white blood cell count.

Parameter	Reference	Day 0	Day 1	Day 2	Day 3	Day 4
Time		22:50	08:15	04:50	17:00	05:30
WBC (×10³/µL)	4.5-11.0	20.86^H^	17.86^H^	-	13.47^H^	11.77^H^
Neutrophils (%)	34-71	85.8^H^	83.0^H^	-	79.6^H^	75.6^H^
ANC (×10³/µL)	1.8-7.7	17.89^H^	14.82^H^	-	10.73^H^	8.90^H^
Immature granulocytes (%)	≤0.4	0.7^H^	0.8^H^	-	2.7^H^	2.5^H^
Lymphocytes (%)	20-50	4.3^L^	7.3^L^	-	11.5^L^	12.1^L^
Hemoglobin (g/dL)	13.0-17.0	8.5^L^	8.1^L^	-	9.1^L^	8.3^L^
Hematocrit (%)	40–52	25.1^L^	23.9^L^	-	27.7^L^	25.9^L^
Platelets (×10³/µL)	150-450	417	429	-	531^H^	454^H^
ESR (mm/hr)	0–15	-	-	106^H^	-	-

**Figure 3:**
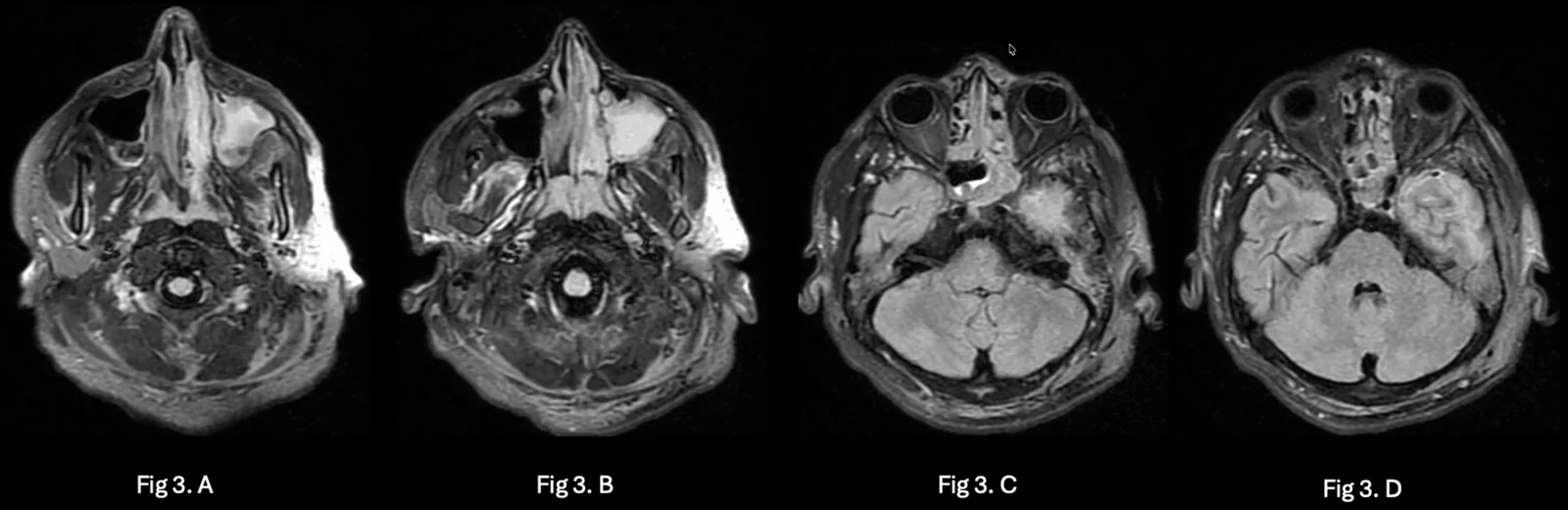
Axial T2-weighted FLAIR MRI demonstrating left-sided sinus opacification and left temporal edema. Axial T2-weighted images and FLAIR magnetic resonance imaging of the brain and paranasal sinuses across four sequential levels, obtained on admission following transfer (A)-(D). Hyperintense signal fills the left maxillary sinus with adjacent inflammatory soft-tissue signal (A) and (B). At the level of the orbits and upper pons, hyperintense signal is present within the left sphenoid sinus with contiguous collection in the left middle cranial fossa. The hyperintense signal also encompasses the left ethmoid sinus with visible cerebral edema in the left temporal region (C) and (D). FLAIR: fluid-attenuated inversion recovery.

**Figure 4:**
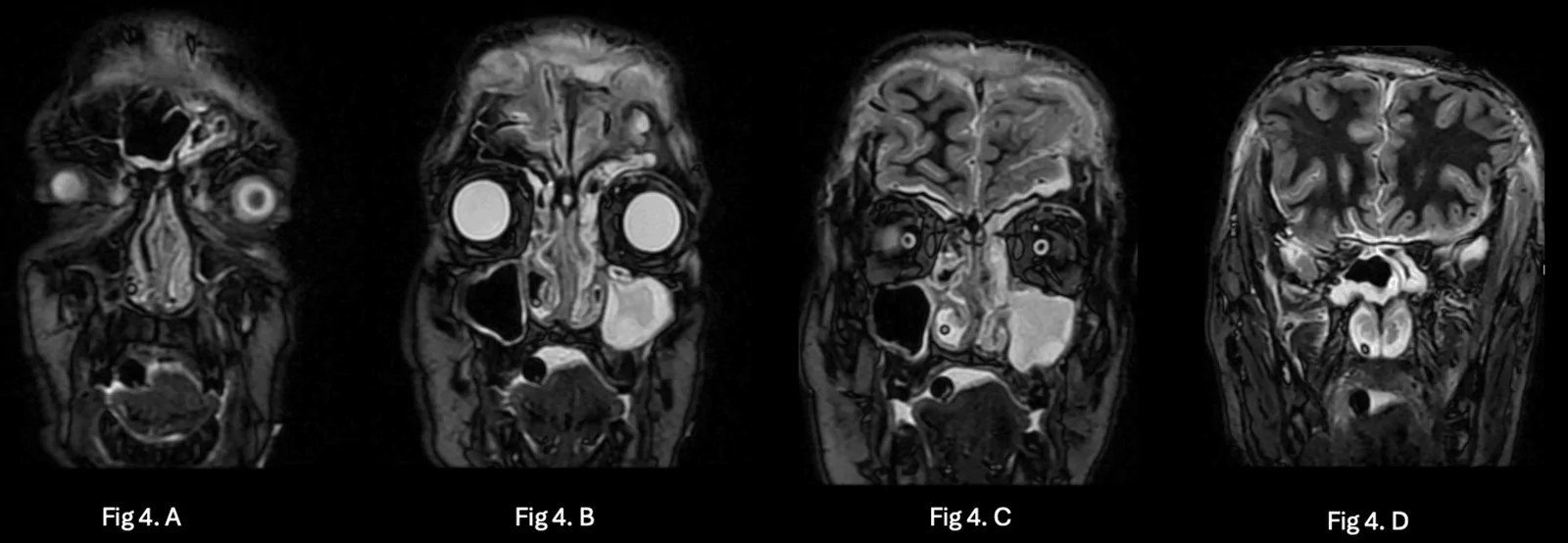
Coronal T2-weighted STIR MRI demonstrating left maxillary and ethmoid sinus hyperintensities with soft tissue involvement. Coronal T2-weighted short tau inversion recovery (STIR) sequences of the paranasal sinuses and orbits across four sequential levels, progressing from anterior to posterior (A)-(D). The most anterior section demonstrates hyperintense signal within the anterior ethmoid air cells and left nasal cavity (A). At the level of the globes, a hyperintense signal is present within the left maxillary sinus along with associated mucosal thickening and hyperintense signal within the left ethmoid air cell (B) and (C). Adjacent hyperintense signal is also present in the periantral soft tissues, consistent with extrasinus inflammatory extension. The right maxillary sinus remains intact and aerated throughout. The most posterior section demonstrates continued left sphenoid sinus and adjacent middle cranial fossa involvement (D). Fat suppression on STIR accentuates the inflammatory fluid signal relative to the surrounding marrow and soft tissue.

The following day, neurosurgery and otorhinolaryngology performed bifrontal craniotomies, anterior and posterior ethmoidal, and maxillary sinusotomies, with bifrontal craniotomies for evacuation of the multifocal subdural empyema. In addition, the patient underwent dural reconstruction with a vascularized pericranial graft in order to provide both structural and immunologic support to the region. Intraoperative cultures grew *Staphylococcus aureus*, *Staphylococcus epidermidis*, *Streptococcus constellatus*, and *Streptococcus intermedius*. Both staphylococcal isolates were methicillin-susceptible, with oxacillin testing confirming ceftriaxone sensitivity. The *Streptococcus* isolates were ceftriaxone- and erythromycin-resistant but susceptible to cefotaxime. This indicated that the streptococcal penicillin-binding proteins remained susceptible to the cephalosporin class; cefepime was therefore selected, providing reliable cephalosporin coverage with a broader spectrum than ceftriaxone. Despite optimizing the dose to 1.4 g IV every 6 hours, vancomycin failed to achieve therapeutic CNS levels, and the regimen was changed to linezolid 600 mg IV every 12 hours to maintain activity and achieve deeper CNS penetration against methicillin-susceptible staphylococcal isolates. Metronidazole was continued for its empiric anaerobic coverage, given the polymicrobial nature of the patient’s sinogenic intracranial infections and the lack of laboratory information on anaerobic susceptibility. The final treatment regimen consisted of cefepime 2 g IV every 8 hours, linezolid 600 mg IV every 12 hours, and metronidazole 500 mg IV every 8 hours, which was continued for a total of 59 days. 

The patient remained in our pediatric intensive care unit for 15 days. On postoperative day 1, he began to exhibit purposeful movement on the right side but then developed a generalized tonic-clonic seizure that was treated with levetiracetam, which was continued for the remainder of the admission. Despite showing initial improvement, the patient’s mental status subsequently declined, requiring intubation; imaging demonstrated postoperative cerebral edema, necessitating bifrontal and left parietal decompressive craniectomies given the patient's altered mental status, Glasgow Coma Scale of 9 and a persistent frontal collection with a newly observed midline shift on imaging (Figure 5). He was extubated after 11 days but had a remaining alteration in mental status, which was treated with risperidone 0.5 mg. 

**Figure 5:**
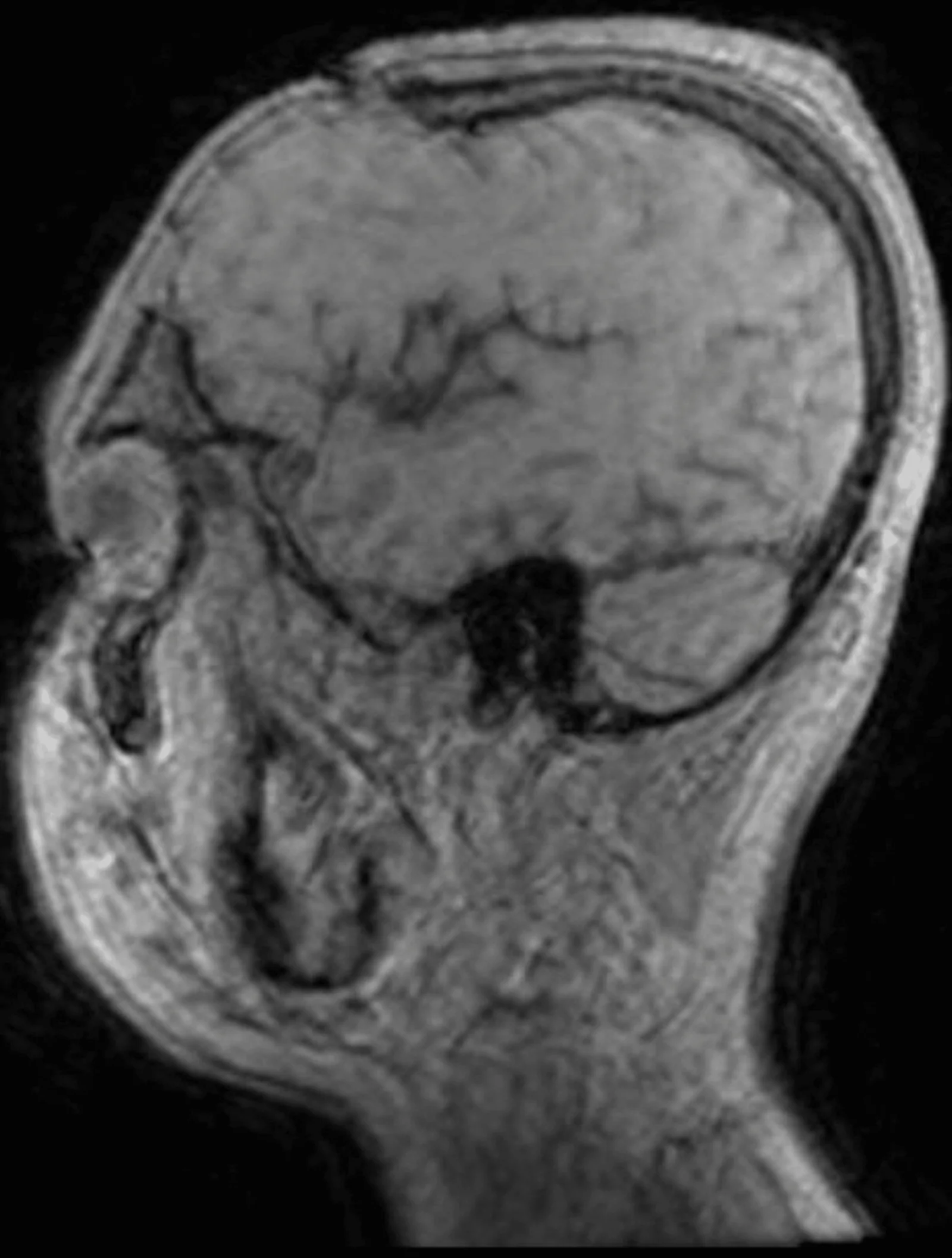
Sagittal T1-weighted post-contrast MRI demonstrating left decompressive craniectomy with herniation of brain parenchyma. Sagittal T1-weighted post-contrast magnetic resonance imaging obtained following decompressive left frontal craniectomy performed for management of postoperative edema. There is a large left-sided defect in the calvarium with outward displacement of brain parenchyma, consistent with expected herniation through the craniectomy site. The underlying parenchyma demonstrates preserved sulci without focal signal abnormalities.

By day 18 of antibiotic therapy, the patient's mental status began to stabilize, with a consistent decline in agitation and recovery of memory, ambulation, and speech. At discharge, the patient had intact memory and coherent speech, a return to his baseline. His laboratory findings showed resolution of leukocytosis with normalization of the neutrophil differential (Table 2). He was scheduled for outpatient neurosurgical follow-up for delayed cranioplasty, given extensive frontal bone necrosis requiring debridement during the acute admission.

**Table 2:** Hematologic trends during the convalescent phase of hospitalization (hospital days 33 to 57). Hematologic laboratory trend during the convalescent phase of hospitalization (hospital days 33–57). Resolution of leukocytosis with normalization of the neutrophil differential is demonstrated. Mild persistent leukopenia with intermittent neutropenia (absolute neutrophil count nadir 1.12 × 10³/µL) was observed during prolonged linezolid therapy and monitored with weekly complete blood counts. Hemoglobin recovered progressively throughout the convalescent period. The erythrocyte sedimentation rate remained mildly elevated, consistent with ongoing resolution of intracranial infection. H, above reference range; L, below reference range; “-”, not measured. ANC, absolute neutrophil count; ESR, erythrocyte sedimentation rate; WBC, white blood cell count.

Parameter	Reference	Day 33	Day 38	Day 43	Day 50	Day 57
Time		04:30	05:30	04:55	05:42	06:40
WBC (×10³/µL)	4.5-11.0	4.46 ᴸ	3.85^L^	3.27^L^	3.57^L^	3.60^L^
Neutrophils (%)	34-71	54.8	43.4	34.2	46.7	49.1
ANC (×10³/µL)	1.8-7.7	2.44	1.67^L^	1.12^L^	1.67^L^	1.77^L^
Immature granulocytes (%)	≤0.4	0.2	0.5^H^	0.3	0.3	0.6^H^
Lymphocytes (%)	20-50	27.4	34.8	45.6	30.3	31.4
Hemoglobin (g/dL)	13.0-17.0	9.2^L^	10.7^L^	11.1^L^	11.0^L^	11.9^L^
Hematocrit (%)	40-52	27.8^L^	32.1^L^	33.7^L^	32.8^L^	35.3^L^
Platelets (×10³/µL)	150-450	197	222	291	342	320
ESR (mm/hr)	0-15	-	-	17^H^	37^H^	32^H^

## Discussion

Sinogenic subdural empyema occupies a uniquely dangerous niche at the intersection of rhinology, neurosurgery, infectious disease, and pediatric critical care. It complicates fewer than 5% of acute pediatric sinusitis cases yet accounts for a disproportionate share of intracranial morbidity in the adolescent age group [1,2,6]. The present case recapitulates each of the canonical features described in contemporary series: an adolescent host, frontal-ethmoid origin, neurologic deterioration disproportionate to early imaging burden, and multifocal pus distribution along the bifrontal convexity, the parafalcine surface, and the left temporal subdural compartment [3,8].

Three anatomic considerations explain why adolescents are uniquely vulnerable. First, the frontal sinus undergoes its principal pneumatization between ages seven and fifteen, leaving an immature and richly vascularized mucosa abutting a thin posterior table [7,16]. Second, the diploic veins of Breschet, which traverse the frontal cortex and communicate with both the dural venous sinuses and the subcutaneous veins, provide a low-resistance corridor for septic thrombophlebitis and retrograde dissemination of bacteria into the subdural space without a frank osseous defect [8,9]. Third, the subdural compartment lacks fibrous attachments to constrain pus collection; once pathogens cross the inner table or seed via emissary veins, infection can spread rapidly along the falx and convexity [3,4]. The result is a fulminant course in which mass effect, cortical irritation, and venous congestion conspire to produce encephalopathy within 24 to 48 hours, often with deceptively limited initial radiographic findings.

The radiologic distinction between subdural empyema and epidural abscess has direct surgical implications. Subdural empyema is typically crescentic, conforms to the cortical surface, crosses suture lines, may extend into the interhemispheric fissure, and demonstrates restricted diffusion on diffusion-weighted imaging (DWI) with rim enhancement on contrast-enhanced T1 sequences [10,12]. Epidural abscess, in contrast, is lentiform, may displace dura inward, and rarely crosses cranial sutures. CT, especially without intravenous contrast, is insensitive to thin subdural collections; pooled estimates suggest a sensitivity of 50% to 70% for empyemas under 5 mm in thickness, compared with greater than 95% for MRI with multiparametric imaging [11,12]. In our patient, the initial 4 mm collection was identified on outside hospital CT, but its true significance and the multifocality of the disease became apparent only on subsequent contrast-enhanced MRI. This pattern, in which CT meaningfully underestimates a neurosurgically actionable empyema, is well-documented and supports the recommendation that any pediatric or adolescent patient with sinusitis and altered mental status undergo urgent multiparametric MRI rather than CT alone [6,12].

The therapeutic principle is twofold: drainage of the subdural compartment and elimination of the originating sinus focus. Drainage technique remains an area of nuanced clinical judgment. Burr hole evacuation is faster and less invasive but yields higher rates of reaccumulation and reoperation, particularly when collections are loculated, parafalcine, or contain thick purulence [11,13]. Craniotomy permits wider exposure, microsurgical lysis of subdural adhesions, and copious irrigation of the parafalcine and basal compartments. Comparative pediatric series suggest that craniotomy is preferable for multifocal or interhemispheric collections, whereas burr-hole drainage may be appropriate for unilocular convexity disease in patients with preserved mental status [4,13,14]. Equally important is the recognition that some patients require staged operative management. Our patient underwent an initial bifrontal craniotomy with evacuation of multifocal collections and dural reconstruction using a vascularized pericranial graft, followed by a bifrontal and left temporal decompressive craniotomy when serial imaging, clinical symptoms, and intracranial pressure trends demonstrated progressive cerebral edema and a previously occult left temporal subdural collection. In addition, some of the frontal bone had to be debrided as it had begun to undergo necrosis due to the intracranial infection, for which a custom frontal bone plate would be designed after complete recovery. Osteomyelitis of the frontal bone is a known complication of advanced pansinusitis with intracranial involvement [7,8]. Delayed cranioplasty is consistent with established algorithms of treatment and infection resolution prior to implant placement [15]. The need for delayed decompressive craniotomy in pediatric empyema, although uncommon, is increasingly recognized in cases with extensive cerebritis or cortical venous compromise and should not be interpreted as therapeutic failure [13]. Concurrent endoscopic source control of the offending sinusitis is associated with shorter duration of intravenous antibiotic therapy and lower rates of reoperation [6,9,16]; accordingly, otolaryngology performed left frontal sinusotomy, anterior and posterior ethmoidectomy, and bilateral maxillary antrostomies during the index operation, deliberately leaving the posterior table of the frontal sinus undisturbed to avoid contamination of the subdural field. 

Empiric antimicrobial coverage targeted the polymicrobial flora characteristic of paranasal sinus infection, including *Streptococcus anginosus *group organisms, anaerobes such as *Fusobacterium* and *Prevotella*, and beta-lactamase-producing aerobes; vancomycin, ceftriaxone, and metronidazole remain a defensible empiric regimen until culture-directed narrowing is feasible [17,18]. The leukocytosis of 20.86 × 10^9^/L with neutrophilic predominance, elevated ESR, and isolated microscopic hematuria without pyuria observed in our patient are common but non-specific accompaniments of severe pyogenic intracranial infection and reinforce the value of imaging-based, rather than serologic, diagnosis. Moreover, changing from ceftriaxone to cefepime and adding linezolid were key to eradicating ceftriaxone-resistant *Streptococci*, especially given their capacity to penetrate the CNS. These agents’ high CNS bioavailability and bacteriostatic activity against Gram-positive organisms at intracranial sites made them a key component of therapy. 

Neuropsychiatric symptoms following severe intracranial infection in adolescents have been described in recent years; these may persist past the resolution of an acute infection. In this case, post-extubation agitation with persistent alteration of mental status required low doses of risperidone [19,20]. These symptoms resolved over a period of weeks with continued antibiotic therapy, physical therapy, psychiatric treatment, and other forms of rehabilitative support. This highlights the importance of prompt interdisciplinary involvement in cases of pediatric CNS infections.

Outcomes have improved markedly in the era of cross-sectional imaging and modern surgical and antimicrobial therapy. Established negative prognostic factors include preoperative obtundation, delay from symptom onset to surgery beyond 72 hours, cortical venous thrombosis, and multilobar parenchymal involvement [4,8,13]. Yet a subset of patients, including ours, present with profound encephalopathy and multifocal disease, require staged neurosurgical intervention with decompressive craniotomy, and nevertheless recover to baseline neurologic function. Several mechanisms may contribute. Early operative decompression appears to interrupt secondary injury cascades before irreversible cortical damage develops; the immature pediatric brain retains a remarkable capacity for synaptic reorganization after inflammatory insult; and timely antimicrobial control may limit cytokine-mediated and venous thrombotic injury beyond the primary lesion [9,17,18]. The discordance between presentation and outcome in such cases suggests that current prognostic models, weighted toward admission Glasgow Coma Scale, age, and imaging burden, may be incomplete and overly deterministic. The biology of recovery, no less than the biology of injury, deserves dedicated investigation in adolescent neuroinfectious disease.

## Conclusions

Pediatric sinogenic subdural empyema remains a time-critical neurosurgical emergency in which a favorable outcome could be determined less by the severity of the initial presentation than by the rapidity and coordination of recognition, imaging, and surgery. The present case demonstrates that an adolescent with multifocal disease and depressed consciousness, even when requiring staged operative intervention with a bifrontal and left temporal decompressive craniotomy for refractory cerebral swelling, combined left frontal, anterior and posterior ethmoidal, and maxillary sinusotomies, can return to neurologic baseline when multiparametric MRI is obtained early, when broad-spectrum antimicrobial therapy is begun without delay, and when neurosurgical evacuation is performed concurrently with endoscopic source control of the offending sinus. Severe presentation should not be equated with poor prognosis, and the determinants of pediatric neurologic recovery from intracranial infection deserve dedicated investigation. The case is offered as both an illustration of optimal multidisciplinary care and a hypothesis-generating example of the resilience of the developing brain.
